# AKT/GSK3β signaling pathway is critically involved in human pluripotent stem cell survival

**DOI:** 10.1038/srep35660

**Published:** 2016-10-20

**Authors:** Leonardo Romorini, Ximena Garate, Gabriel Neiman, Carlos Luzzani, Verónica Alejandra Furmento, Alejandra Sonia Guberman, Gustavo Emilio Sevlever, María Elida Scassa, Santiago Gabriel Miriuka

**Affiliations:** 1 Laboratorios de Investigación Aplicada en Neurociencias (LIAN-CONICET), Fundación FLENI, Ruta 9, Km 52.5, Escobar, Buenos Aires, B1625XAF, Argentina; 2 Laboratorio de Regulación de Expresión Génica, IQUIBICEN, UBA/CONICET, Dptos. de Química Biológica y de Fisiología, Biología Molecular y Celular, Facultad de Ciencias Exactas y Naturales, Universidad de Buenos Aires, Intendente Güiraldes 2160, Buenos Aires, C1428EGA, Argentina

## Abstract

Human embryonic and induced pluripotent stem cells are self-renewing pluripotent stem cells (PSC) that can differentiate into a wide range of specialized cells. Basic fibroblast growth factor is essential for PSC survival, stemness and self-renewal. PI3K/AKT pathway regulates cell viability and apoptosis in many cell types. Although it has been demonstrated that PI3K/AKT activation by bFGF is relevant for PSC stemness maintenance its role on PSC survival remains elusive. In this study we explored the molecular mechanisms involved in the regulation of PSC survival by AKT. We found that inhibition of AKT with three non-structurally related inhibitors (GSK690693, AKT inhibitor VIII and AKT inhibitor IV) decreased cell viability and induced apoptosis. We observed a rapid increase in phosphatidylserine translocation and in the extent of DNA fragmentation after inhibitors addition. Moreover, abrogation of AKT activity led to Caspase-9, Caspase-3, and PARP cleavage. Importantly, we demonstrated by pharmacological inhibition and siRNA knockdown that GSK3β signaling is responsible, at least in part, of the apoptosis triggered by AKT inhibition. Moreover, GSK3β inhibition decreases basal apoptosis rate and promotes PSC proliferation. In conclusion, we demonstrated that AKT activation prevents apoptosis, partly through inhibition of GSK3β, and thus results relevant for PSC survival.

Human embryonic stem cells (hESCs) were described more than 10 years ago when Thomson and colleagues published the methodology for isolating and maintaining pluripotent stem cells (PSC) in culture in an undifferentiated state for several passages^1^. From this discovery, many laboratories demonstrated that these cells have a high *in vitro* potency to differentiate into any type of cell (except those that form a placenta or embryo), a property called pluripotency. In recent years the field was further advanced by Yamanaka and colleagues with a new way of obtaining PSC that are very similar to embryonic cells, the so-called human induced pluripotent stem cells (hiPSCs)^2^. Potentially, these cells may then be a plausible cell source for regenerative medicine, and are regularly used in *in vitro* models for the study of human development, diseases and drug discovery. Hence, an intense research in many areas is currently conducted in the field.

PSC are in a delicate balance between survival, self-renewal, differentiation and death. Culture conditions are critical for sustaining any of these possible outcomes. Various signaling pathways activated through fibroblast growth factor receptor (FGFR) are involved in cell proliferation, differentiation and apoptotic processes in many different cell types^3^. Among them are undifferentiated PSC, which express high levels of several FGF family members, including receptors and ligands^4^,^5^. Indeed, it has been demonstrated that basic fibroblast growth factor (bFGF) is essential for PSC stemness and self-renewal maintenance, and most laboratories relies on the use of bFGF for maintaining the surviving pluripotent state^4^,^6^,^7^,^8^,^9^. However, it is now understood that these culture conditions are suitable for human epiblastic pluripotent stem cells propagation, but more stringent conditions are necessary to turn and keep cells in a higher level of undifferentiation, usually called *naïve* PSC.

In particular, Phosphatidylinositol 3-kinase (PI3K) signaling pathway, a known regulator of cell survival and proliferation in different cellular contexts, is activated by bFGF^3^,^10^,^11^. A very well characterized target of PI3K is AKT, also known as protein kinase B. Once activated, AKT can phosphorylate downstream substrates such as BAD and Caspase-9 and thereby promote cell survival^10^. It has been reported that PI3K/AKT activation by bFGF is relevant to maintain the undifferentiated state of hESCs^12^. Moreover, it was found that inhibition of FGF receptors with SU5402 diminishes AKT phosphorylation/activation levels and induces hESCs differentiation^13^.

hESCs and hiPSCs present a high *in vitro* rate of spontaneous apoptosis and nonspecific differentiation. Therefore, human PSC expansion is difficult and inefficient^1^,^14^,^15^,^16^. For example, it has been reported that up to 30% of hESCs grown in standard media conditions undergo spontaneous apoptosis^15^,^17^,^18^. Moreover, almost 40% of hESCs differentiate spontaneously after 12 days of *in vitro* culture^19^. Considering that the culture system for PSC is based on the addition of bFGF and insulin to promote cell survival, PI3K/AKT role in hESCs survival is still controversial. Armstrong *et al*. reported no significant differences in hESCs apoptosis rate upon PI3K inhibition with selective inhibitor LY294002^20^. However, other studies demonstrated that PI3K activity may be important for hESCs survival^12^,^13^,^16^,^21^,^22^. Hence, PI3K/AKT signaling is critical for the maintenance of the undifferentiated state of PSC, but also could be relevant in regulating PSC survival.

In the present study we explored the relevance and molecular mechanisms involved in the regulation of hESCs and hiPSCs survival by AKT. We found that pharmacological inhibition of AKT with three non-structurally related inhibitors (GSK690693, AKT inhibitor VIII and AKT inhibitor IV) decreased cell viability and increased phosphatidylserine (PS) translocation to the outer leaflet of the plasma membrane, DNA fragmentation, Caspase-9 cleavage, Caspase-3 activation and PARP proteolysis in hESC lines WA01 (H1) and WA09 (H9) and in a hiPSCs cell line generated in our laboratory (FN2.1). Moreover, no relevant changes in BCL-2 family members BCL-X_L_, BCL-2 and BAX protein products were found. Importantly, Glycogen synthase kinase β (GSK3β) signaling seems to be responsible, at least in part, of the apoptosis induction observed upon AKT inhibition. Remarkably, GSK3β inhibition also decreased basal apoptosis rate and induced proliferation of undifferentiated PSC maintained on standard cell culture conditions. Therefore, we conclude that PI3K/AKT/GSK3β signaling prevents programmed cell death and is relevant to guarantee PSC survival.

## Results

### Specific pharmacological AKT inhibitors impair AKT activity in human PSC

**Figure 1:**
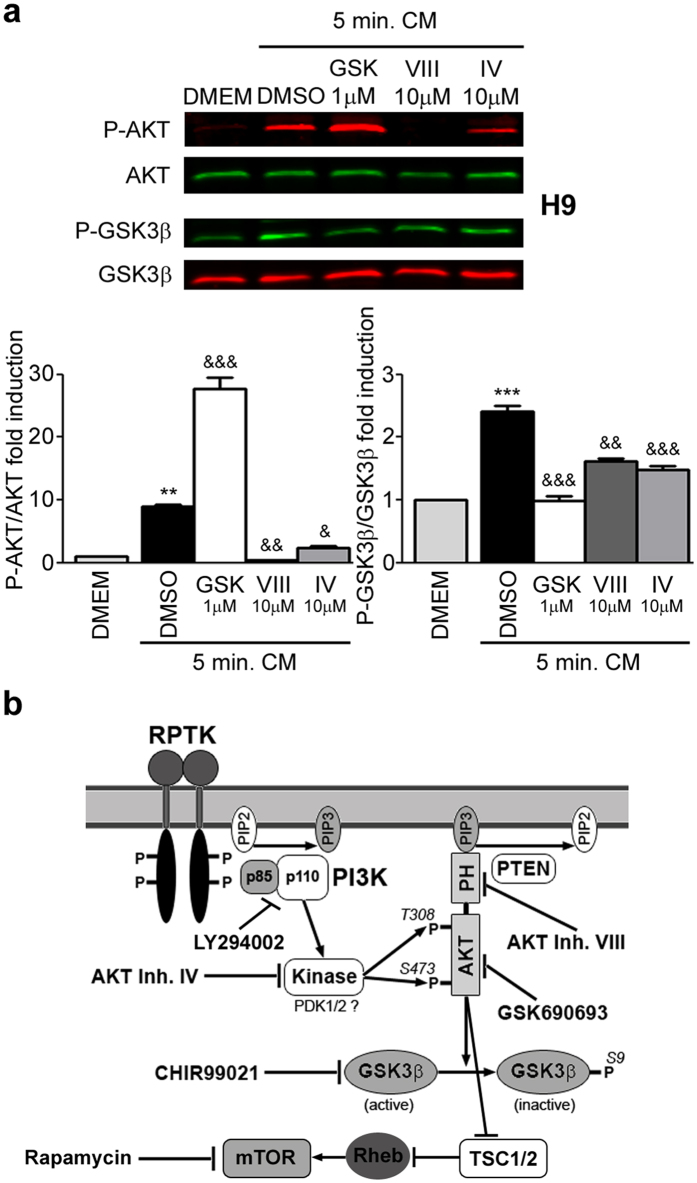
AKT phosphorylation and activity status. (**a**) H9 hESCs grown on Matrigel were starved for 6 hours with KSR/bFGF-free DMEM/F12 cell culture medium and then changed for 5 minutes to complete iMEFs conditioned medium (CM) supplemented with 8 ng/ml bFGF plus DMSO (Vehicle) or any AKT inhibitor [GSKi (GSK, 1 μM), AKTi VIII (VIII, 10 μM) and AKTi IV (IV, 10 μM)]. After the starvation/stimulation period, p-AKT (Ser473), AKT, p-GSK3β (Ser9) (p-AKT substrate) and GSK3β expression levels were analyzed and quantified by Western blots with IR fluorescence secondary antibodies and Odyssey Imagers in order to test inhibitors efficacy in human pluripotent stem cells. The bars represent the level of p-AKT/AKT and p-GSK3β/GSK3β fold induction relative to untreated starved cells. The mean + SEM from three independent experiments are shown. Statistical analysis was performed by one-way ANOVAs followed by Tukey’s multiple comparisons test, **p < 0.01 and ***p < 0.001 vs. DMEM; ^&^*p *< 0.05; ^&&^*p *< 0.01 and ^&&&^*p*< 0.001 vs. DMSO. **(b)** Schematic drawing of the PI3K/AKT/GSK3β and mTOR signaling pathway. PI3K is activated through receptor-binding tyrosine kinases (RPTK) by growth factors (as bFGF) resulting in phosphorylation of PIP2. PIP3 subsequently acts as a second messenger allowing the binding of Pleckstrin homology (PH) domain-containing proteins like AKT. Thereby the latter undergoes conformational changes leading to its phosphorylation and activation by PDK1/2. Termination of the signaling cascade can either occur through the dephosphorylation of PIP3 or AKT by PTEN or PP2A phosphatases, respectively. AKT participates in the regulation of cellular processes like cell growth and apoptosis by phosphorylating further proteins, such as GSK3β or TSC1/2 (which leads to mTOR activation). The target sites on the PI3K/AKT/GSK3β and mTOR signaling pathway of each of the inhibitors tested (GSK3β inhibitor CHIR99021; mTOR inhibitor Rapamycin; PI3K inhibitor LY294002; AKT specific inhibitors VIII, IV and GSK690693) is shown.

We first analyzed the activation status of AKT and GSK3β by sensitizing the PSC cell culture condition with a starvation/stimulation protocol. Phosphorylation levels of AKT and GSK3β were evaluated and quantified by Western blot at the end of starvation (6 hours treatment with DMEM/F12 culture media deprived of KSR and bFGF) and immediately after stimulation [5 minutes *treatment with* iMEF conditioned media (CM) supplemented with bFGF] periods. Figure 1a shows that stimulation induced a rapid increase in the amount of phosphorylated AKT at Serine 473 and its substrate GSK3β at Serine 9 [8.91 ± 0.31 and 2.41 ± 0.10 fold induction vs. DMEM/F12 for p-AKT (Ser473) and p-GSK3β (Ser9), respectively] (lanes 1 and 2, first and third rows, respectively, and graph).

We then tested, under these experimental conditions, the effect of three non-structurally related AKT specific pharmacological inhibitors on AKT activity. All these inhibitors act at different sites of AKT signaling pathway. The inhibitors used were: GSK690693 (GSKi) (potent and selective, ATP-competitive, pan-AKT kinase inhibitor)^23^, AKT inhibitor VIII (AKTi VIII) (binds the Pleckstrin Homology domain of AKT1/2 isoenzymes and prevents binding of AKT to cell membrane)^24^,^25^ and AKT inhibitor IV (AKTi IV) (targets the ATP-binding site of a kinase upstream of AKT and downstream of PI3K)^26^ (Fig. 1b). We observed that AKTi VIII and IV were able to strongly restrain AKT phosphorylation and activity (evidenced by analysis of GSK3β phosphorylation) induced by CM [(AKTi VIII: 0.037 ± 0.002 and 0.67 ± 0.006 fold induction vs. CM + DMSO, for p-AKT and p-GSK3β, respectively); (AKTi IV: 0.19 ± 0.10 and 0.62 ± 0.05 fold induction vs. CM + DMSO, for p-AKT and p-GSK3β, respectively)] (Fig. 1a, lanes 2, 4 and 5, first and third panels, respectively and graph). On the other hand, AKT inhibitor GSKi significantly impaired AKT activity (0.41 ± 0.07 P-GSK3β fold induction vs. CM + DMSO) but not its phosphorylation level. Importantly, AKT phosphorylation was strongly induced upon GSKi treatment (3.11 ± 0.10 p-AKT fold induction vs. CM + DMSO) (Fig. 1a, lanes 2 and 3, first and third panels, respectively and graph).

### AKT inhibition induces apoptosis of human PSC

**Figure 2:**
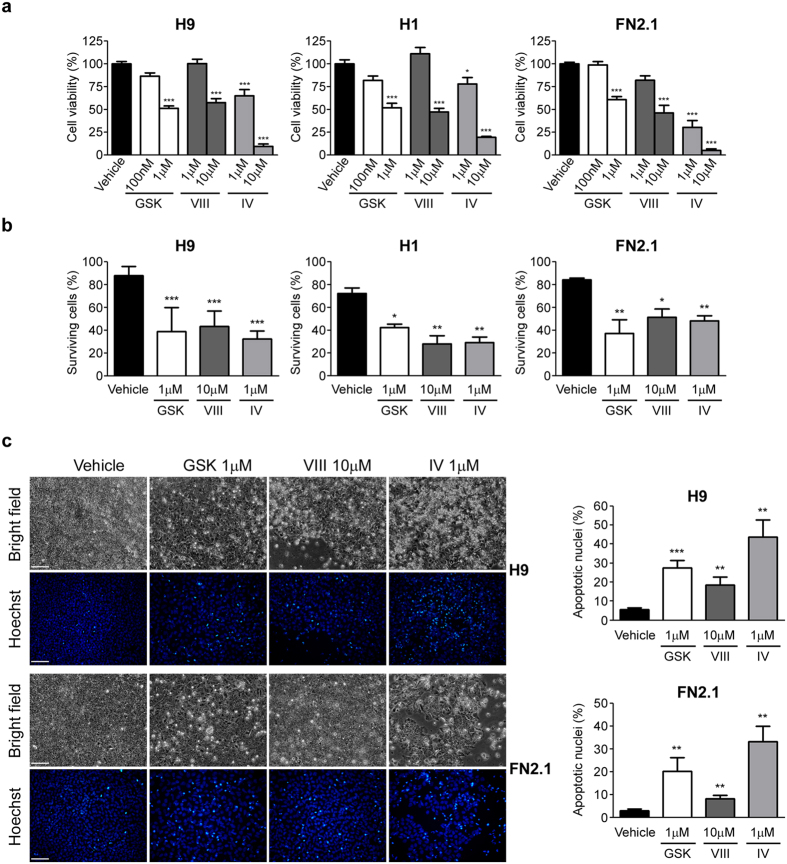
hESCs and hiPSCs cell viability upon AKT inhibitors treatment. (**a**) H9, H1 hESCs and FN2.1 hiPSCs cell viability was analyzed 24 hours post-treatment with increasing concentrations of AKTi IV (IV), AKTi VIII (VIII) and GSKi (GSK) by XTT colorimetric assay. Vehicle = DMSO. Mean + SEM from three independent experiments are shown. Statistical analysis was done by one-way ANOVAs followed by Tukey’s multiple comparisons test, *p < 0.05 and ***p < 0.001 vs. Vehicle. **(b)** Histogram shows percentage of surviving cells assessed by Trypan blue exclusion method 24 hours after incubation with AKT inhibitors [AKTi IV (IV, 1 μM), AKTi VIII (VIII, 10 μM) and GSKi (GSK, 1 μM)]. Mean + SEM from at least three independent experiments are shown. Statistical analysis was done by one-way ANOVAs followed by Tukey’s multiple comparisons test, *p = <0.05; **p = <0.01 and ***p = <0.001 vs. Vehicle (DMSO). **(c)** Chromatin condensation was analyzed by Hoechst staining 24 hours after incubation of H9 and FN2.1 cells with AKT inhibitors [AKTi IV (IV, 1 μM), AKTi VIII (VIII, 10 μM) and GSKi (GSK, 1 μM)]. Figure shows representative images and means + SEM from three independent experiments are graphed for % of apoptotic nuclei. The scale bar represent 100 μm. Statistical analysis was done by Student’s t-test, **p = <0.01 and ***p = <0.001 vs. Vehicle (DMSO).

We next evaluated how AKT specific pharmacological inhibition affected hESCs (H9 and H1) and hiPSCs (FN2.1) viability. We determined the percentage of cell viability after 24 hours of treatment with increasing concentrations of GSKi, AKTi VIII and AKTi IV using a XTT/PMS vital dye assay. As shown in Fig. 2a, cell viability felt down significantly in all tested cells lines (H9, H1 and FN2.1) with all tested AKT inhibitors. As expected, changes in cell viability were concentration dependent and concentrations that reduced cell viability to near 50% were chosen for further experiments (1 μM for GSKi, 10 μM for AKTi VIII and 1 μM for AKTi IV). Similar results were obtained when live and dead cells were counted using Trypan blue dye-exclusion assay. As shown in Fig. 2b the percentage of surviving cells markedly decreased 24 hours after AKT inhibitors addition. Interestingly, we observed that 11.9 ± 2.7%, 27.8 ± 3.4% and 15.8 ± 0.8% of H9, H1 and FN2.1 cells grown on Matrigel coated surfaces undergo spontaneous cell death, respectively (Fig. 2b, vehicle treatment).

To analyze whether the reduction in cell viability observed upon AKT inhibition is related to apoptosis induction we evaluated the appearance of apoptotic features in hESCs (H9 and H1) and hiPSCs (FN2.1). Chromatin condensation paralleled by ballooning and cell detachment are some of the criteria which are used to identify apoptotic cells. Therefore we next measured these morphological changes by Hoechst staining of nuclear DNA and bright field images of treated and control cells, respectively. We observed that AKT inhibition (24 hours treatment with AKTi VIII 10 μM, AKTi IV 1 μM and GSKi 1 μM) increased the percentage of hESCs and hiPSCs Hoechst positive apoptotic nuclei (5.5 ± 0.6% Vehicle, 27.4 ± 2.2% GSKi 1 μM, 18.6 ± 2.3% AKTi VIII and 43.7 ± 5.1% AKTi IV for H9 cells; 2.9 ± 0.4% Vehicle, 20.2 ± 3.4% GSK 1 μM, 8.2 ± 0.8% AKTi VIII and 33.2 ± 3.8 AKTi IV for FN2.1 cells) as well as cell detachment and ballooning (Fig. 2c and graph).

As previously mentioned, AKT is a well characterized target of PI3K (Fig. 1b). We then confirmed if PI3K activity is relevant for human PSC survival as it was previously reported^12^,^13^,^16^,^21^. Importantly, we impaired PI3K activity with the pharmacological inhibitor LY294002 (10 and 30 μM) on hESCs and hiPSCs and observed a strong inhibition of AKT phosphorylation, a decrease of cell viability (by XTT/PMS vital dye assay), a reduction on the percentage of surviving cells (by Trypan blue dye-exclusion assay), an increase of late apoptosis or necrosis rate (by flow cytometry analysis with PI staining) and an increment on the percentage of apoptotic nuclei (by Hoechst staining of nuclear DNA) (see Supplementary Fig. S1).

**Figure 3:**
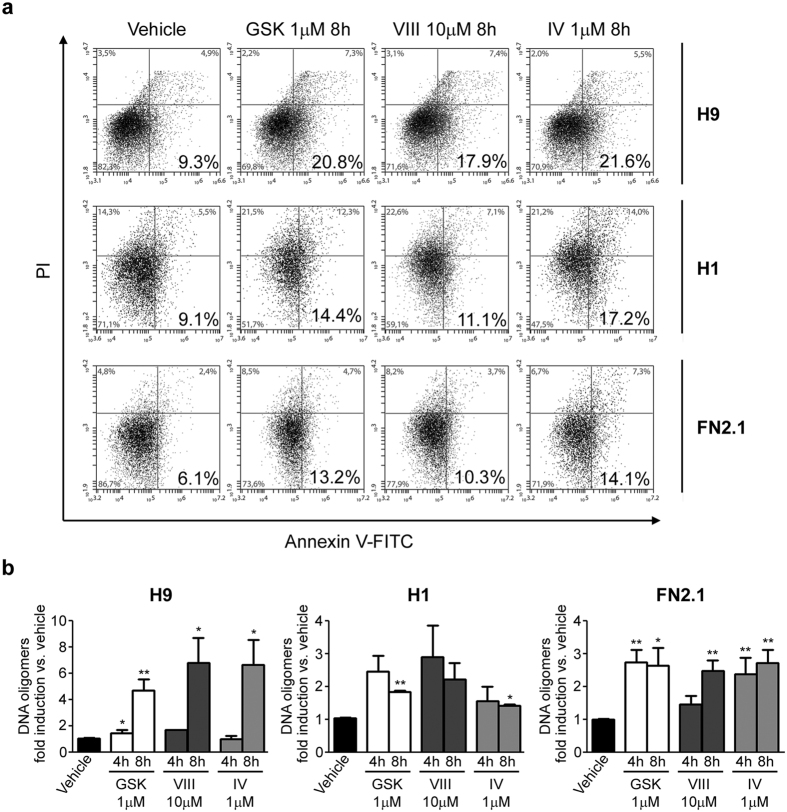
Annexin V translocation and DNA fragmentation upon AKT inhibition. (**a**) Phosphatidylserine (PS) translocation from the inner to the outer leaflet of the plasma membrane was examined by Annexin V and propidium iodide (PI) double staining. A representative of three independent experiments biparametric flow cytometry analysis of combined fluorescein isothiocyanate (FITC)-conjugated Annexin V and PI staining distinguishing viable (PI^−^, Annexin V^−^ bottom left), early apoptotic (PI^−^, Annexin V^+^ bottom right), late apoptotic (PI^+^, Annexin V^+^; top right) and necrotic (PI^+^, Annexin V^−^, top left) cells is shown for H9, H1 and FN2.1 after 8 hours of incubation with AKT inhibitors [AKTi IV (IV, 1 μM), AKTi VIII (VIII, 10 μM) and GSKi (GSK, 1 μM)]. Percentage of cells in each quadrant is shown. (**b**) Genomic DNA fragmentation into oligomers of 180–200 bp or multiples of that was quantified in H9, H1 and FN2.1 cells at 4 and 8 hours post-treatment with AKT inhibitors [AKTi IV (IV, 1 μM), AKTi VIII (VIII, 10 μM) and GSKi (GSK, 1 μM)] using a specific ELISA kit. Mean + SEM fold induction relative to Vehicle (DMSO) of three independent experiments are shown. Statistical analysis was done by Student’s t-test, *p = <0.05 and **p = <0.01 vs. Vehicle (DMSO).

Phosphatidylserine (PS) translocation from the inner to the outer leaflet of the plasma membrane has been considered an early feature of apoptosis. Therefore, we examined PS exposure and plasma membrane integrity simultaneously by Annexin V, a phospholipid-binding protein with high affinity for PS, and propidium iodide (PI) double staining on PSC treated with AKT specific pharmacological inhibitors. PI can only penetrate the plasma membrane when membrane integrity is breached, as occurs in the later stages of apoptosis or in necrosis. By flow cytometry analysis we observed an increased number of Annexin V^+^/PI^−^ apoptotic cells after 8 hours AKT inhibition with the three pharmacological inhibitors tested (GSKi 1 μM, AKTi VIII 10 μM and AKTi IV 1 μM), concomitant with a decrease in the number of live cells (Annexin V^−^/PI^−^). The number of Annexin V^+^/PI^+^ necrotic cells also increased during the timeframe of the experiments (Fig. 3a). To further explore if the decrease of cell viability was a consequence of AKT inhibition-induced apoptosis, we measured DNA fragmentation (cytoplasmic oligonucleosomal fragments of approximately 180–200 bp, or multiples of that, representative of inter-nucleosomal cleavage of DNA), a late event in the apoptotic signaling pathway^27^. Therefore we quantified DNA oligomers with an immunoassay, using antibodies directed against DNA and histones. As shown in Fig. 3b, a marked increase in the proportion of DNA oligomers was observed at 4 and 8 hours upon AKT specific inhibitors treatment in H9, H1 and FN2.1 cells.

**Figure 4:**
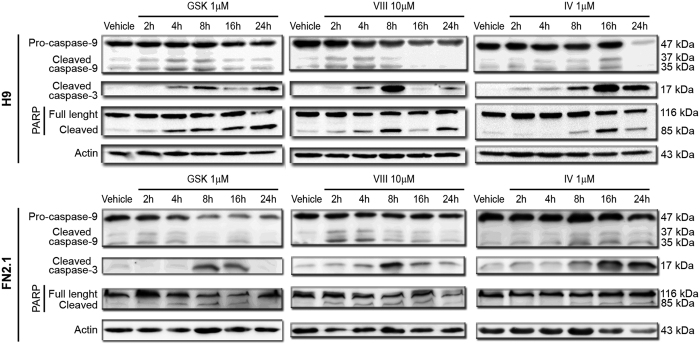
Caspase-9, Caspase-3 activation and PARP cleavage upon AKT inhibitors incubation. Cleavage and activation of initiator Caspase-9, effector Caspase-3 and PARP proteolysis (Caspase-3 substrate) were analyzed by Western blot in H9 and FN2.1 cells at 2, 4, 8, 16 and 24 hours post specific AKT inhibitors treatment [AKTi IV (IV, 1 μM), AKTi VIII (VIII, 10 μM) and GSKi (GSK, 1 μM)]. Actin was used as loading control. Representative blots of three independent experiments are shown.

Caspases, a family of endoproteases, play an essential role in programmed cell death. Once activated, caspases cleave diverse substrates in the cytoplasm or nucleus. Thus, activation of initiator and effector caspases is another relevant criterion to determinate apoptosis induction. Upon AKT inhibition, initiator pro-Caspase-9 (47 kDa) was cleaved into active fragments (37/35 kDa). A decrease in pro-Caspase-9 and an increase in active fragments levels were detected by Western blot in H9 and FN2.1 cells (Fig. 4). Cleaved Caspase-9 could further process other caspase members (effector caspases), including Caspase-3, to initiate caspase cascade that leads to apoptosis. Western blot detection of cleaved Caspase-3 (appearance of Caspase-3 p17) revealed a time-dependent activation of Caspase-3, concomitant with Caspase-9 activation, mediated by AKT inhibition (Fig. 4). In parallel, time course studies showed the presence of cleaved PARP (effector caspases substrate) which was preceded by the appearance of the catalytically active form p17. This chronology is compatible with the involvement of Caspase-3 in PARP proteolysis (Fig. 4). Interestingly, we determined that AKT inhibition resulted in the activation of apoptotic caspases with very similar kinetics in hiPSCs FN2.1 and hESCs H9. However, caspases activation was delayed in time in both FN2.1 and H9 cells when AKTi IV was used at the tested concentration.

Taken together, these results indicate that specific AKT inhibition using three non-structurally related inhibitors induces apoptosis of human PSC. Based on the presence of cleaved Caspase-9, the mitochondrial-mediated apoptosis pathway participates in this induction.

### AKT inhibition does not influence BCL-2 family members expression levels

**Figure 5:**
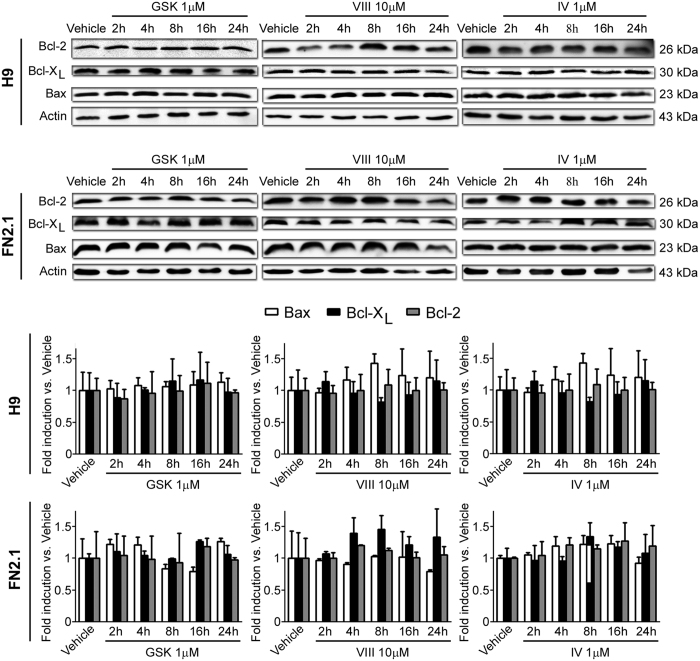
BCL-2 family member expression levels. Expression levels of BCL-2 family members, including BAX (pro-apoptotic), BCL-2 (anti-apoptotic) and BCL-X_L_ (anti-apoptotic) were analyzed by Western blot in H9 and FN2.1 cells at 2, 4, 8, 16 and 24 hours post AKT inhibitors treatment [AKTi IV (IV, 1 μM), AKTi VIII (VIII, 10 μM) and GSKi (GSK, 1 μM)]. Actin was used as loading control. Mean + SEM fold induction relative to Vehicle (DMSO) and representative blots of three independent experiments are shown.

The apoptotic threshold is governed by the subcellular localization and abundance of pro- and anti-apoptotic proteins. Thus, to determine the expression levels of key regulators of apoptosis in hESCs H9 and hiPSCs FN2.1 after AKT inhibition for 2, 4, 8, 16 and 24 hours with the specific inhibitors tested, we measured BCL-2 and BLC-X_L_ (anti-apoptotic proteins) and BAX (pro-apoptotic factor) abundance by Western blot. Results shown in Fig. 5 indicate that neither anti-apoptotic proteins BCL-2 and BCL-X_L_ nor the pro-apoptotic protein BAX significantly changed their expression levels at different time points upon AKT inhibition.

### Involvement of mTOR and GSK3β signaling in AKT regulation of hESCs and hiPSCs cell viability and apoptosis

Mammalian target of rapamycin (mTOR) is a downstream effector of AKT and has been indicated as a central regulator of cell metabolism, growth, proliferation and survival^28^. On this sense it has been proposed that mTOR signaling could mediate cell survival in stem cells^22^. In order to test if mTOR could be involved in AKT regulation of PSC viability and survival we evaluated the effect of the highly selective mTOR inhibitor Rapamycin (Fig. 1b) on cell viability and apoptosis induction of PSC. We observed a significant decrease in both H9 and FN2.1 cell viability using a XTT/PMS vital dye assay at 24 hours post Rapamycin (10 and 100 nM) treatment. However, inhibition of mTOR did not significantly reduced the percentage of surviving cells (by Trypan blue dye-exclusion assay) neither increased late apoptosis or necrosis rate (by flow cytometry analysis with PI staining) nor the percentage of apoptotic nuclei (by Hoechst staining of nuclear DNA). Only a slight induction of cell death was observed with 100 nM Rapamycin (see Supplementary Fig. S2). Importantly, the efficacy of Rapamycin was corroborated by the appearance of autophagic features in glioma stem cells (see Supplementary Fig. S3). In conclusion, mTOR signaling is not critical for PSC survival regulation, even though we cannot totally rule out a minor role.

**Figure 6:**
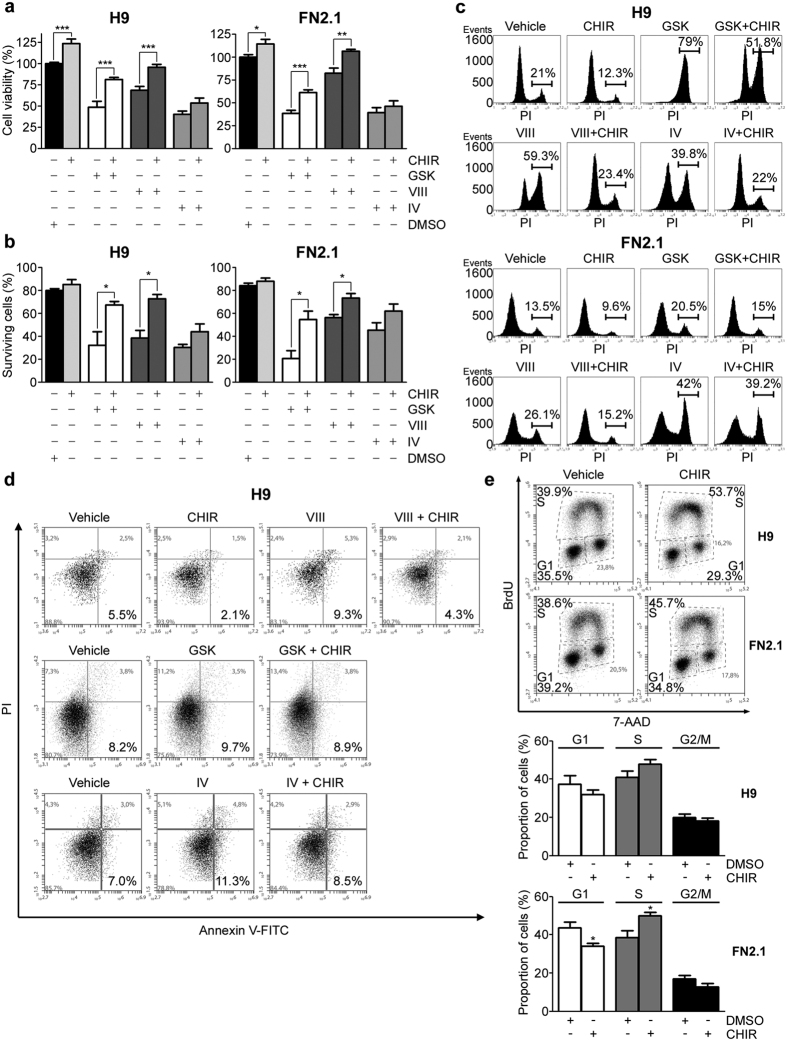
Involvement of GSK3β signaling in AKT regulation of hESCs and hiPSCs cell viability and apoptosis. (**a**) H9 and FN2.1 cell viability was analyzed by XTT colorimetric assay at 24 hours post-treatment with AKT inhibitors IV (IV, 1 μM), VIII (VIII, 10 μM) and GSKi (GSK, 1 μM) in the presence or absence of CHIRi (CHIR, 3 μM). Mean + SEM from three independent experiments are shown. Statistical analysis was performed by Student’s t test, *p = <0.05; **p = <0.01 and ***p = <0.001 (**b**) Histogram shows quantitative percentage of surviving cells assessed by Trypan blue exclusion method 24 hours post-AKT inhibitors treatment [IV (1 μM), VIII (10 μM) and GSK (1 μM)] with or without CHIRi (CHIR, 3 μM). Mean + SEM from three independent experiments are shown. Student’s t test, *p = <0.05. (**c**) Representative histograms, of three independent experiments, of Propidium iodide (PI) staiStatistical analysis was donened H9 and FN2.1 unfixed cells treated for 24 hours with AKT inhibitors [IV (1 μM), VIII (10 μM) and GSK (1 μM)] in combination or not with CHIRi (CHIR, 3 μM). Percentage of PI positive cells (late apoptotic or necrotic) was determined by flow cytometric analysis. Vehicle: DMSO. (**d**) A representative biparametric flow cytometry analysis, of three independent experiments, of combined fluorescein isothiocyanate (FITC)-conjugated Annexin V and PI staining identifying viable (bottom left), early apoptotic (bottom right), late apoptotic (top right) and necrotic (top left) cells is shown for H9 cells at 8 hours post-AKT inhibitors treatment [IV (1 μM), VIII (10 μM) and GSK (1 μM)] in combination or not with CHIRi (CHIR, 3 μM). Vehicle: DMSO. Percentage of cells in each quadrant is shown. (**e**) Representative BrdU-APC/7-AAD flow cytometry cell cycle analysis of H9 and FN2.1 undifferentiated cells treated with CHIRi (CHIR, 3 μM) for 24 hours. Vehicle: DMSO. Means + SEM from three independent experiments are graphed for the proportion of cells (%) in each stage of cell cycle (G1, S and G2/M). Statistical analysis was performed by Student’s t-test, *p = <0.05 vs. Vehicle (DMSO).

On the other hand, GSK3β is a multifunctional serine/threonine kinase which has been implicated in multiple biological processes including embryonic development, cell differentiation, proliferation, apoptosis and insulin responsiveness^29^,^30^. GSK3β phosphorylation at Serine 9, which can be mediated by AKT, has an inhibitory effect on GSK3β activity. Therefore, AKT inhibition could lead to an increase in GSK3β activity (Fig. 1b). As we mentioned, GSK3β phosphorylation at Serine 9 is impaired by AKT specific inhibitors GSKi, AKTi VIII and AKTi IV (Fig. 1a). It is then possible that GSK3β signaling could be involved in AKT regulation of hESCs and hiPSCs viability and survival. To test this hypothesis, we evaluated the effect of the highly selective GSK3β inhibitor CHIR99021 (CHIRi) (Fig. 1b) on cell viability and apoptosis induction triggered by AKT inhibition. We first determined the percentage of cell viability using a XTT/PMS vital dye assay 24 hours after treatment with GSKi (1 μM), AKTi VIII (10 μM) and AKTi IV (1 μM) with or without CHIRi (3 μM). As shown in Fig. 6a, the decreasing cell viability effect of AKT inhibition in both H9 and FN2.1 was partially reverted by CHIRi in all cases. Interestingly, GSK3β inhibition enhanced cell viability of untreated undifferentiated H9 and FN2.1 cells (DMSO treated cells). Similar results were obtained when cells were quantified using Trypan blue dye-exclusion assay. As shown in Fig. 6b the percentage of surviving cells, which markedly decreased 24 hours after AKT inhibition, was partially reverted by CHIRi treatment.

To analyze whether the effects triggered by GSK3β inhibition in cell viability were related to the reversion of apoptosis induced by AKT inhibition, we measured late apoptosis or necrosis by flow cytometry analysis with PI staining in H9 and FN2.1 cells. The histograms in Fig. 6c show the percentages of treated (AKT inhibitors in combination or not with CHIRi) or untreated cells exhibiting loss of plasma membrane integrity (late apoptosis or necrosis). Again, GSK3β inhibition with CHIRi (3 μM) decreased apoptosis/necrosis levels in control untreated cells and partially reverted apoptosis/necrosis induction caused by AKT inhibitors. To further demonstrate that GSK3β is involved in AKT inhibition mediated apoptosis induction we performed an Annexin V/PI double staining assay. We observed that the increased number of Annexin V^+^/PI^−^ cells (early apoptosis) observed after 8 hours of AKT inhibition with GSKi (1 μM), AKTi VIII (10 μM) and AKTi IV (1 μM) was reverted by the concomitant inhibition of GSK3β with CHIRi (3 μM). Again, CHIRi (3 μM) treatment reduced basal apoptosis rate in control undifferentiated PSC maintained in standard cell culture conditions (Fig. 6d).

As GSK3β has been also implicated in proliferation, we wondered if the effect of GSK3β inhibition by CHIRi in hESCs and hiPSCs basal apoptosis rate was associated with an increase in the proliferation rate. We then evaluated the effect of GSK3β inhibition on cell cycle profiles in undifferentiated PSC (H9 and FN2.1) growing on Matrigel coated dishes with CM. Cells were treated with CHIRi (3 μM) for 24 hours and flow cytometry performed with dual labeling of cells with 7-AAD and anti-BrdU-APC. Interestingly, GSK3β inhibition caused an increase in the proportion of cells in the synthesizing S phase and a decrease in the number of cells in the G1 phase of the cell cycle in both cell lines, which implies a raise on the proliferation rate (Fig. 6e and graph).

However, as some reports suggest that the effect of GSK3β inhibitors or Wnt-3a may vary according to cell culture conditions (e.g. undefined iMEF CM)^15^, we repeated most of the above mentioned experiments culturing human PSC on Vitronectin coated dishes in combination with fully defined Essential 8 (E8) medium. Again, the effect of AKT inhibition in decreasing cell viability and on apoptosis/necrosis induction in both H9 and FN2.1 cells was partially reverted by GSK3β inhibition with CHIRi. Interestingly, the effect of AKT inhibition on cell viability and apoptosis/necrosis induction was even stronger when H9 hESCs were cultured in defined E8 medium. Besides, GSK3β inhibition enhanced cell viability of H9 and FN2.1 untreated undifferentiated cells (see Supplementary Fig. S4).

**Figure 7:**
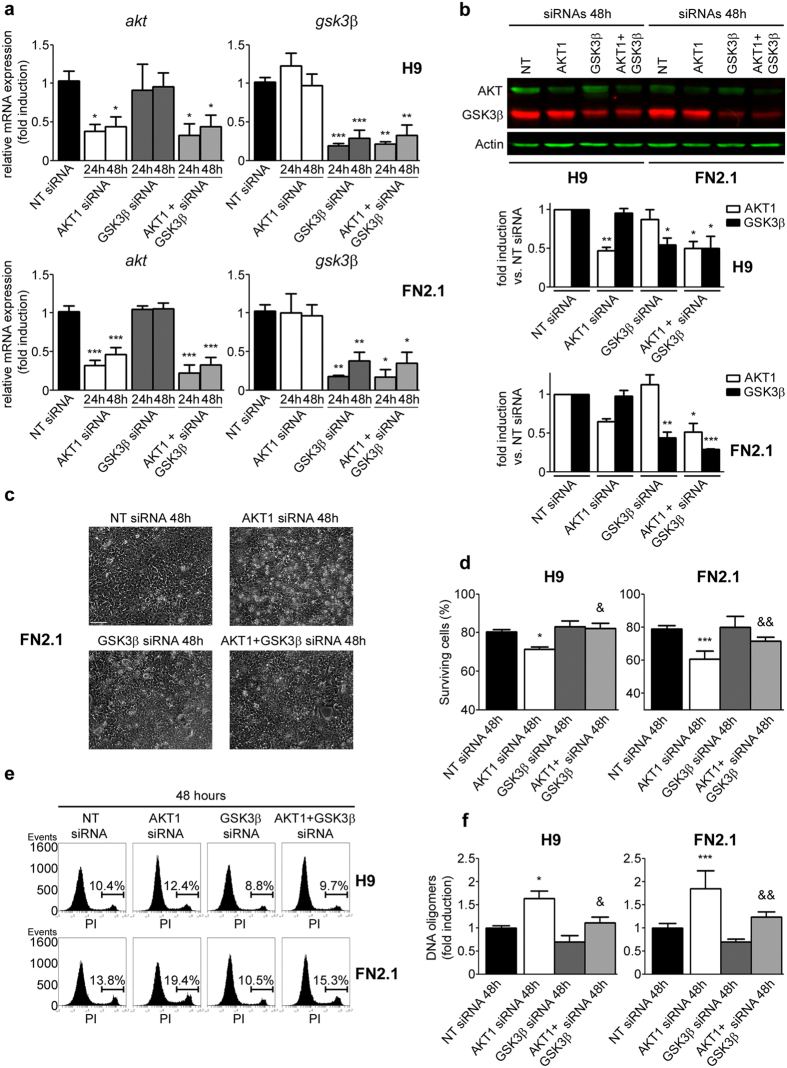
Effect of siRNA-mediated down regulation of AKT1 and GSK3β in hESCs and hiPSCs cell viability and apoptosis. H9 hESCs and FN2.1 hiPSCs grown until 85% confluence with E8 media in Vitronectin coated dishes were transfected with negative control no-targeting siRNA (NT siRNA) (10 nM) or AKT1 siRNA (10 nM) or GSK3β siRNA (10 nM) or AKT1 + GSK3β siRNAs (10 nM) and then: (**a**) mRNA expression levels of *akt* and *gsk3β* were analyzed by Real Time RT-PCR at 24 and 48 hours post siRNAs transfection. *rpl7* expression was used as normalizer. Graph shows mean + SEM mRNA fold induction relative to NT siRNA transfectants arbitrarily set as 1 from three independent experiments. (**b**) Expression levels of AKT and GSK3β were analyzed by Western blot in H9 and FN2.1 cells at 48 hours post siRNAs transfection. Actin was used as loading control. Mean + SEM fold induction relative to the corresponding NT siRNA and representative blots of three independent experiments are shown. (**c**) Representative images of FN2.1 cells at 48 hours post siRNAs transfection are shown. The scale bars represent 100 μm. (**d**) Histograms show percentage of surviving cells assessed by Trypan blue exclusion method 48 hours post siRNAs transfection. Mean + SEM from five independent experiments are shown. (**e**) Representative histograms, of three independent experiments, of Propidium iodide (PI) stained H9 and FN2.1 unfixed cells at 48 hours post siRNA transfection. Percentage of PI positive cells (late apoptotic or necrotic) was determined by flow cytometric analysis. (**f**) Genomic DNA fragmentation into oligomers of 180–200 bp or multiples of that was quantified in H9 and FN2.1 cells at 48 hours post siRNAs transfection using a specific ELISA kit. Mean + SEM fold induction relative to NT siRNA of four independent experiments are shown. (**a**,**b**,**d**,**f**) Statistical analysis was done by one-way ANOVAs followed by Tukey’s multiple comparisons test, ***p <0.001; **p <0.01 and *p <0.05 vs. NT siRNA; ^&&^*p* < 0.01 and ^&^*p *< 0.05 vs. AKT1 siRNA.

Finally, in order to confirm AKT/GSK3β axis involvement on human PSC apoptosis, we used siRNA knockdown to silence either AKT or GSK3β or both kinases. In all cases siRNA mediated knockdown was assessed by RT-qPCR and Western blot in hESCs (H9) and hiPSCs (FN2.1) cultured in defined E8 medium and transfected with either non-targeting control siRNA (nt-siRNA) or specific siRNAs. As shown in Fig. 7a,b, siRNA transfection led to a significant decrease in AKT and/or GSK3β mRNA and protein levels. Under the same experimental conditions, we found that siRNA-mediated downregulation of AKT, at 48 hours post-transfection, induced ballooning and cell detachment, reduced the percentage of surviving cells (by Trypan blue dye-exclusion assay) and increased late apoptosis or necrosis (by flow cytometry analysis with PI staining) and apoptotic DNA fragmentation (by DNA oligomers quantification by ELISA) rates (Fig. 7c–f, respectively). As expected, the above mentioned processes were not affected by siRNA-mediated downregulation of GSK3β, except, and in concordance with previously mentioned results, for some reduction in basal (comparing with nt-siRNA treated cells) late apoptosis or necrosis and DNA fragmentation rates (Fig. 7e,f). Besides, and importantly, the effect of AKT knockdown was partially reverted when AKT and GSK3β were simultaneously silenced on human PSC (Fig. 7c–f).

Taken together, the above results suggest that GSK3β signaling is, at least in part, responsible of the apoptotic induction caused by AKT inhibition in human PSC. Moreover, GSK3β is involved in the high spontaneous apoptosis rate observed in hESCs and hiPSCs, and its inhibition increases PSC proliferation rate.

## Discussion

PSC need to keep their genome integrity as they have the ability to differentiate into all cell types of the three germ layers, endoderm, mesoderm and ectoderm. As a consequence, hESCs and hiPSCs are highly sensitive to exogenous insults and rapidly trigger apoptosis rather than repair the damaged genome^31^,^32^,^33^.

Gaining insights into the mechanisms of apoptosis regulation in PSC results relevant to overcome one of the greatest obstacles that faces regenerative medicine which is the potential of introducing non-desired undifferentiated teratoma-forming cells during transplantation of differentiated cells. Therefore, the understanding of why human PSC are so sensitive to external cues could help to improve a large-scale *in vitro* expansion of PSC with optimized cell culture conditions, the generation of a safe transplantable cell source with no or minimal risk for tumor/teratoma formation and the clinical outcome of therapies relying human pluripotent-derived cells^33^,^34^.

As previously mentioned, signaling pathways activated by bFGF are essential for human PSC pluripotency and self-renewing^4^,^6^,^7^,^8^. PI3K/AKT activation by bFGF has been demonstrated to be important for the maintenance of the undifferentiated state of hESCs^12^,^35^. However, the role of PI3K/AKT signaling pathway on hESCs survival or apoptosis regulation is controversial as there are opposing reports on this regard^12^,^13^,^16^,^20^,^21^,^22^. All the above mentioned reports have been focused on PI3K kinase activity, consequently we directed our attention on its most studied downstream target, AKT, whose role in cell survival and apoptosis prevention is very well documented in many different cellular contexts^36^,^37^. For this purpose, we used three structurally-unrelated AKT pharmacological inhibitors: GSK690693 (GSKi)^23^, AKT inhibitor VIII (AKTi VIII)^24^,^25^ and AKT inhibitor IV (AKTi IV)^26^ to specifically inhibit AKT activity on several PSC lines. We were able to demonstrate that at commonly used concentrations these three reagents were able to effectively impair AKT activity, as judged by the phosphorylation status of a major substrate, GSK3β. Interestingly, as previously mentioned, even though AKT activity was inhibited with GSKi, its phosphorylation status was strongly conserved. This AKT hyperphosphorylation could be due to the occupancy of the ATP binding pocked of AKT by GSKi, which restricts phosphatases access and further sustains AKT phosphorylation^38^,^39^. Importantly, under these experimental conditions we found that AKT specific inhibition with the three tested compounds decreased cell viability and induced apoptosis, defined by increased PS translocation to the outer leaflet of the plasma membrane, DNA fragmentation, Caspase-9 cleavage, Caspase-3 activation and PARP proteolysis in human PSC. Therefore, we can conclude that PI3K/AKT signaling is anti-apoptotic and thus relevant in promoting hESCs and hiPSCs survival.

In relation to the molecular mechanisms involved, we focused our attention on two of the downstream effectors of AKT with previously reported implications in cell survival regulation in many different cellular contexts: mTOR and GSK3β^28^,^29^,^30^. In the case of mTOR, its pharmacological inhibition with Rapamycin did not significantly affected PSC basal apoptosis rate, in concordance with previous reports^21^. Although a decrease in cell viability measured using the XTT/PMS assay was observed upon mTOR inhibition, probably due to the already known role of mTOR signaling in PSC proliferation^40^. These results suggest that mTOR signaling is not critical for PSC cell survival, even though we cannot discard some degree of participation. On the other hand, our findings demonstrated that GSK3β inhibition with CHIR99021 (CHIRi) partially reverted AKT inhibition-induced apoptosis in H9 hESCs and FN2.1 hiPSCs. Importantly, we also proved AKT/GSK3β involvement on human PSC cell apoptosis by siRNA knockdown approach. Phosphorylation of Serine 9 inhibits GSK3β, whereas de-phosphorylation of this residue activates it^41^. Normally, GSK3β activity is suppressed by proliferative, pro-survival signals that increase Serine 9 phosphorylation, such as WNT ligands, EGF, IGF-I and -II, and bFGF, as well as AKT^41^,^42^,^43^,^44^. We observed that GSK3β phosphorylation in Serine 9 is impaired by AKT specific inhibitors rendering this kinase inactive. It is also known that the most common apoptotic pathways are the intrinsic ones, which are mediated by the mitochondria. Paradoxically, it was reported that GSK3β promotes the cell death caused by the mitochondrial intrinsic apoptotic pathway, but inhibits the death receptor-mediated extrinsic apoptotic pathway^30^. This could be also the case for human PSC. We observed an increased level of Caspase-9 cleavage, which implies that the mitochondrial-mediated apoptosis pathway may participate in the apoptosis induced by AKT inhibition/GSK3β activation.

As previously mentioned, human PSC are very susceptible to undergo programmed cell death. Moreover, they present a high *in vitro* rate of spontaneous apoptosis^15^,^17^,^18^. Mechanisms behind this apoptosis-prone state remain elusive. Recent reports suggested that hESCs sensitivity to apoptotic stimuli may be linked to their high state of mitochondrial priming, probably determined by their pro- and anti-apoptotic proteins expression profiles^45^. It is then conceivable that in PSC cells apoptosis could be controlled by post-translational modifications rather than changes in protein expression levels. This situation would allow a rapid apoptotic response that will only require activation of kinase cascades to induce apoptosis. Minor changes in the environment could potentially result in rapid cell death, necessary to prevent the propagation of mutations during the early critical stages of embryonic development^34^. Intriguingly, GSK3β directly phosphorylates and activates BAX on Serine 163^46^. In addition, GSK3β has been implicated in promoting mitochondrial permeabilization by direct phosphorylation and destabilization of MCL-1. As mentioned before, MCL-1 is an anti-apoptotic member of the BCL-2 family highly expressed in hESCs and hiPSCs^47^,^48^. Importantly, we observed that GSK3β inhibition with CHIRi reduced hESCs and hiPSCs basal apoptotic rates and increased proliferation. In this sense, and in accordance with our results, it was recently demonstrated that, during reprogramming of murine somatic cells, inhibition of GSK3β completely rescues cell survival and proliferation rate blocked by the AKT inhibitor MK2206^49^. Interestingly, the opposite effect was reported on mouse embryonic stem cells, where GSK3β inhibition with CHIRi at similar concentrations resulted to be cytotoxic^50^. One possible mechanism that may explain why PSC have a high *in vitro* rate of spontaneous apoptosis or how AKT inhibition induces PSC apoptosis is BAX activation and/or MCL-1 destabilization mediated by GSK3β. This could lead to a rapid translocation of BAX to mitochondria or to a change in the balance of pro- and anti-apoptotic proteins that would induce the mitochondrial outer-membrane permeabilization and in consequence the mitochondrial intrinsic apoptotic pathway activation.

In conclusion, we demonstrated that AKT signaling is anti-apoptotic in both hESCs and hiPSCs. Moreover, GSK3β signaling mediates in part the apoptotic induction observed upon AKT inhibition, although we cannot rule out that other pathways are involved in this process. Importantly, GSK3β inhibition by CHIR99021 reduced basal apoptosis rate and induced proliferation in all the human PSC lines tested. These findings should be taken in consideration in the optimization of human PSC cell culture conditions, especially for cell lines that present higher *in vitro* rate of spontaneous apoptosis. Moreover, the effect of GSK3β inhibition in apoptosis regulation should be specially studied in protocols that use CHIR99021 or similar for the generation of human naive pluripotent stem cells^51^ or for reprogramming human somatic cells to hiPSCs^52^.

## Methods

### Cell lines and culture

hESCs lines WA01 (H1) and WA09 (H9)^1^ were purchased from WiCell Research Institute (Madison, WI, USA, http://www.wicell.org) at low passages (p15 to p20). Both cell lines are approved for US National Institute of Health (NIH) funding. hiPSCs line FN2.1 has been previously derived at our laboratory from human foreskin fibroblasts in accordance with relevant guidelines and regulations and was fully validated^33^,^53^,^54^. Moreover, all experimental protocols where hiPSCs line FN2.1 was used, including derivation, were given ethical approval by the local Ethics Committee (Comité de ética en investigaciones biomédicas del Instituto FLENI) and written informed consent was obtained from donor prior to foreskin fibroblast isolation. PSC lines were maintained on an inactivated mouse embryonic fibroblast (iMEF) feeder layer in medium comprised of Dulbecco’s Modified Eagle’s Medium/Ham’s F12 (DMEM/F12) supplemented with 10% Knockout Serum Replacement (KSR), 2 mM nonessential amino acids, 2 mM L-glutamine, 100 U/ml penicillin, 50 μg/ml streptomycin, 0.1 mM β-mercaptoethanol and 4 ng/ml of bFGF. PSC were transferred with 1 mg/ml collagenase IV into feeder-free diluted (1/40) Matrigel (BD Matrigel^TM^ Basement Membrane Matrix, BD Bioscience, San Jose, CA, USA) coated dishes in iMEF conditioned medium (CM). CM was prepared as previously described^55^. Before experiments, PSC grown on Matrigel were dissociated into single cells using Accutase 1x for 20 minutes, plated onto Matrigel coated dishes (with addition of 10 μM Y-27632 ROCK inhibitor) and grown until confluence with CM. For some experiments feeder-free cultures of PSC were maintained on Vitronectin (0.5 μg/cm^2^) coated dishes (VTN-N, Life Technologies, CA, USA) in combination with fully defined Essential 8 medium (E8, Life Technologies, CA, USA). Cultures were split every 3 to 4 days by means of PBS-EDTA (Versene) passaging. Before experiments, PSC grown on Vitronectin and E8 were dissociated into single cells using Accutase 1x for 20 minutes, plated onto Vitronectin coated dishes (with addition of 10 μM Y-27632 ROCK inhibitor) and grown until confluence with E8. All cell lines were free of *Mycoplasma sp.* infection, which was tested as previously described^55^.

### Inhibitors

GSK690693 (generously provided by GlaxoSmithKline, USA); AKT inhibitors VIII, IV and LY294002 (Calbiochem, San Diego, CA, USA); CHIR99021 (Tocris, Bristol, UK); and Rapamycin (Sigma, St. Louis, MO, USA) were dissolved in DMSO and stored at −80 °C protected from light. Inhibitors were added to cell cultures such that the final DMSO concentrations were not higher than 0.10% (v/v).

### Cell viability assay

PSC were plated onto Matrigel coated 96-well plates at densities between 1 × 10^4^–3 × 10^4^ cells per well and grown until confluence. 24 hours post-treatments, 50 μg/well of activated 2,3-bis *(2-methoxy-4-nitro-5-sulfophenyl)-5 [(phenylamino) carbonyl]-2 H-tetrazolium hydroxide* (XTT) in PBS containing 0.3 μg/well of N-methyl dibenzopyrazine methyl sulfate (PMS) were added (final volume 100 μl) and incubated for 1–2 hours at 37 °C. Cellular metabolic activity was determined spectrophotometrically at 450 nm.

### Hoechst staining

PSC were grown until confluence and, 24 hours post-treatments, stained with Hoechst 33342 (2 μg/ml) for 20 minutes. Stained cells were examined under a Nikon Eclipse TE2000-S inverted microscope equipped with a 20X E-Plan objective and a super high-pressure mercury lamp. The images were acquired with a Nikon DXN1200F digital camera, which was controlled by the EclipseNet software (version 1.20.0 build 61). Percentages of apoptotic nuclei were calculated as total number cells showing chromatin condensation divided by total number of cells and multiplied by 100.

### Trypan blue staining

For Trypan blue exclusion assay, PSC were seeded in 6-well tissue culture plates at a density of 1 × 10^5^ cells/ml. At 24 hours post-treatments, adherent and detached cells were collected and stained with 0.4% Trypan blue solution (final concentration 0.08%) for 5 min at room temperature. Cells were counted in a hemocytometer chamber. Percentages of surviving cells (unstained) were calculated as total number of live cells divided by total number of cells (stained) and multiplied by 100.

### Flow cytometric analysis of cell viability by Propidium Iodide (PI) staining

24 hours after incubation with inhibitors, single-cell suspensions were obtained with Accutase treatment (37 °C for 7 minutes). PSC were then centrifuged at 200× g for 5 minutes and resuspended up to 1 × 10^6^ cells/ml in FACS Buffer (2.5 mM CaCl_2_, 140 mM NaCl and 10 mM HEPES pH 7.4). Next, 100 μl of cellular suspension were incubated with 5 μl of PI (50 μg/ml) in PBS for 5 minutes in the dark. Finally, 400 μl of FACS Buffer were added to each tube and cells were immediately analyzed by flow cytometry. Data was acquired on a BD Accuri C6 flow cytometer and analyzed using BD Accuri C6 software.

### Flow cytometric determination of apoptosis by Annexin V/Propidium iodide double staining

FITC-Annexin V Apoptosis Detection Kit I was used to measure cell death by flow cytometry according to manufacturer’s instructions (BD Bioscience, Heidelberg, Germany). Briefly, cells were washed twice with PBS, and then pellets were re-suspended in 1 × Binding Buffer (0.01 M HEPES pH 7.4, 0.14 M NaCl and 2.5 mM CaCl_2_) at a concentration of 1 × 10^6^ cells/ml. Each sample (100 μl of the solution, 1 × 10^5^ cells) was transferred to a tube and was stained with 5 μl Annexin V-FITC and 5 μl PI. After incubation in the dark for 15 minutes at room temperature, 400 μl of 1 × Binding Buffer was added to each tube and cell suspensions were analyzed by flow cytometry within one hour. Data was acquired on a BD Accuri C6 flow cytometer using BD Accuri C6 software.

### Assessment of DNA fragmentation

Apoptosis induction was quantified by direct determination of nucleosomal DNA fragmentation with Cell Death Detection ELISAPlus kit (Roche, Mannheim, Germany) as previously described^33^. Briefly, 2 × 10^5^ PSC were plated on 24-well culture plates in 500 μl cell culture media. Four or eight hours after AKT inhibitors incubation, cells were lysed according to manufacturer’s instructions, followed by centrifugation (200 × g, 5 minutes). The mono and oligonucleosomes in the supernatants were determined using an anti-histone-biotinilated antibody. The resulting color development was measured at 405 nm wavelength using a multiplate spectrophotometer. Results were expressed as DNA oligomer fold induction versus vehicle (DMSO), calculated from the ratio of absorbance of treated samples to that of the untreated ones.

### BrdU staining and flow cytometry

The BrdU-APC flow kit (BD Pharmingen, CA, USA) was used to analyze proliferation of PSC. Briefly, cells growing on Matrigel coated 12-well dishes with CM were pulsed with 10 μM BrdU for 30 minutes. The cells were then fixed, permeabilized, washed, and stained with anti-BrdU-APC and 7-AAD, according to manufacturer’s instructions. The proliferation status of PSC was examined by gating out the BrdU^+^ fraction by a BD Accuri C6 flow cytometer. Flow cytometry data were analyzed using BD Accuri C6 software.

### Protein analysis

Total proteins were extracted from PSC in ice-cold RIPA protein extraction buffer supplemented with protease (Protease inhibitor cocktail set I, Calbiochem, San Diego, CA, USA) and phosphatase (10 mM sodium fluoride and 1 mM sodium orthovanadate) inhibitors. Protein concentration was determined using Bicinchoninic Acid Protein Assay (Pierce™, Rockford, IL, USA). Equal amounts of protein were run on 15% or 10% SDS-polyacrylamide gel electrophoresis and transferred to PVDF or PVDF-FL (Millipore, Billerica, MA, USA) membranes. Blots were blocked 1 hour at room temperature in TBS (20 mMTris–HCl, pH 7.5, 500 mM NaCl) containing low-fat powdered milk (5%) and Tween 20 (0.1%) or with Odyssey blocking buffer (LI-COR Biosciences, Lincoln, NE, USA). Incubations with primary antibodies were performed overnight at 4 °C in blocking buffer (3% skim milk, 0.1% Tween, in Tris-buffered saline) or in Odyssey blocking buffer containing 0.1% Tween 20. The membranes were then incubated with the corresponding counter-antibody and the proteins revealed by enhanced chemiluminescence detection (SuperSignal West Femto System, Thermo Scientific, Rockford, IL, USA); alternatively, antigen/primary antibody complexes were detected with near infrared-fluorescence-labeled secondary antibodies using an Odyssey Infrared Imaging Scanner. For information about antibodies please see Supplementary methods. Densitometric analysis of protein levels were performed with ImageJ 1.34 s software (Wayne Rasband, National Institutes of Health, USA) for chemiluminescence detection. For Licor Odyssey protein quantification, antibody signals were analyzed as the average 700 and 800-channel integrated intensities with Odyssey imaging software 3.0.

### Cell transfection and RNA Interference

Cells were transfected with the corresponding small interfering RNA (siRNA) using Lipofectamine™ RNAiMAX lipid reagent (Invitrogen, CA, USA) as per manufacturer’s instructions. Briefly, 2 × 10^5^ cells were plated unicellular on Vitronectin-coated 24-well dishes, grown 24 hours with E8 media and then transfected with Silencer Select Negative Control #2 (Ambion™, cat#4390846), Silencer Select Validated AKT1 siRNA (Ambion™, Cat. # 4390824, siRNA ID:s659) or Silencer Select Validated GSK3β siRNA (Ambion™, Cat. # 4390824, siRNA ID: s6241) (Invitrogen, CA, USA). The concentration of siRNA used for cell transfection was 10 nM.

### RNA isolation and RT-qPCR

Total RNA was extracted from PSC with Trizol and cDNA was synthesized from 500 ng of total RNA with 15 mM of random hexamers and MMLV reverse transcriptase (Promega, WI, USA), according to manufacturer’s instructions. For real-time PCR studies, cDNA samples were diluted 5-fold and PCR amplification and analysis were performed with StepOnePlus Real Time PCR System (PE Applied Biosystems, CA, USA). The SYBR^®^ GreenER^TM^ qPCR SuperMix UDG (Invitrogen, CA, USA) was used for all reactions, following manufacturer’s instructions. For information about primers sequences please see Supplementary methods.

### Statistical analysis

All results are expressed as mean ± SEM. One-way ANOVAs followed by Tukey’s multiple comparisons tests or two-tailed Student’s t-test were used to detect significant differences (p < 0.05) among treatments as indicated.

## Additional Information

**How to cite this article**: Romorini, L. *et al*. AKT/GSK3β signaling pathway is critically involved in human pluripotent stem cell survival. *Sci. Rep.* **6**, 35660; doi: 10.1038/srep35660 (2016).

## Supplementary Material

Supplementary Information
