# Supplementary material for: AKT/GSK3β signaling pathway is critically involved in human pluripotent stem cell survival
Source: Sci Rep. 2016 Oct 20;6:35660. doi: 10.1038/srep35660 (PMC5071844; doi:10.1038/srep35660)
Supplement: Supplementary Information [file srep35660-s1.pdf]

Supplementary information

Title: *AKT/GSK3β signaling pathway is critically involved in human pluripotent stem cell survival*

Authors: Leonardo Romorini[1,*], Ximena Garate[1], Gabriel Neiman[1], Carlos Luzzani[1], Verónica Alejandra Furmento[1], Alejandra Sonia Guberman[2], Gustavo Emilio Sevlever[1], María Elida Scassa[1] and Santiago Gabriel Miriuka[1,*].

Author affiliations: [1]Laboratorios de Investigación Aplicada en Neurociencias (LIAN-CONICET), Fundación FLENI, Ruta 9, Km 52.5, Escobar, Buenos Aires, B1625XAF, Argentina. [2]Laboratorio de Regulación de Expresión Génica, IQUIBICEN, UBA/CONICET, Dptos. de Química Biológica y de Fisiología, Biología Molecular y Celular, Facultad de Ciencias Exactas y Naturales, Universidad de Buenos Aires, Intendente Güiraldes 2160, Buenos Aires, C1428EGA, Argentina.

*** Corresponding authors:** Leonardo Romorini, e-mail: lromorini@fleni.org.ar; Santiago Miriuka, e-mail: smiriuka@fleni.org.ar

Supplementary methods:

Antibodies and primers used

The following primary antibodies were used: α-AKT (sc-1618); α-p-GSK3β (S9) (sc-373800); α-GSK3β (sc-9166); α-BCL-XL/S (sc-634); α-BAX (sc-493); α-BCL-2 (sc-7382); α-PARP (sc-8007) and α-ACTIN (sc-1616) (all from Santa Cruz Biotechnology, Santa Cruz, CA, USA), α-active Caspase-3 (ab13847) (Abcam Inc., Cambridge, MA, USA), α-p-AKT (S473) (cat. 9271S) and α-Caspase-9 (cat. 9502) (Cell Signaling Technology, Beverly, MA, USA).

As secondary antibodies the following were used: a horseradish peroxidase-conjugated α-rabbit IgG; α-mouse IgG or α-goat IgG; or secondary antibodies from Licor Biosciences (NE, USA) conjugated to IRDye 800CW (goat-anti-mouse-IgG) or to IRDye 680 (goat-anti-rabbit-IgG).

Primers used for RT-qPCR were: *rpl7*; sense, 5´-AATGGCGAGGATGGCAAG-3´; antisense, 5´-TGACGAAGGCGAAGAAGC-3´; *akt*; sense, 5´-TGTGTGGACAGCGAGCGCAG-3´; antisense, 5´-GCCGCCTCTCCATCCCTCCA-3´ and *gsk3β*; sense, 5´- TGGAATCTGCCATCGGGATA-3´; antisense, 5´-ATTGGGTTCTCCTCGGACCA-3´.

Glioma cancer stem cells culture

Glioma cancer stem cell line G02 was gently provided by Guillermo A. Videla Richardson, PhD. G02 line was derived by Dr. Videla Richardson in accordance with relevant guidelines and regulations. Ethical approval was given by the local Ethics Committee (Comité de ética en investigaciones biomédicas del Instituto

FLENI) and written informed consent was obtained from donor. Cells were grown on laminin-coated plates (10µg/ml) in serum-free medium consisting of Neurobasal medium supplemented with B27, N2, 20 ng/ml basic fibroblast growth factor (bFGF), 20 ng/ml epidermal growth factor (EGF), 2 mM L-glutamine, 2 mM non-essential aminoacids, 50 U/ml penicillin/streptomycin, 20 µg/ml bovine pancreas insulin and 75 µg/ml low-endotoxin bovine serum albumin. Cells were routinely grown until confluence, dissociated using Accutase and then splitted 1:2 to 1:3. Medium was replaced every 2-3 days.

Cell transfection with RFP-LC3 construct

G02 cancer stem cells were transfected with RFP-LC3 construct using Lipofectamine 2000 lipid reagent (Invitrogen, CA, USA) as per the manufacturer's instructions. Afterwards cells were treated with Rapamycin (10nM) for 24 hours, fixed, and examined under a Nikon Eclipse TE2000-S inverted microscope equipped with a 40X E-Plan objective and a super high-pressure mercury lamp. The images were acquired with a Nikon DXN1200F digital camera, which was controlled by the EclipseNet software (version 1.20.0 build 61).

Supplementary figures:

PI3K inhibition diminishes hESCs and hiPSCs cell viability

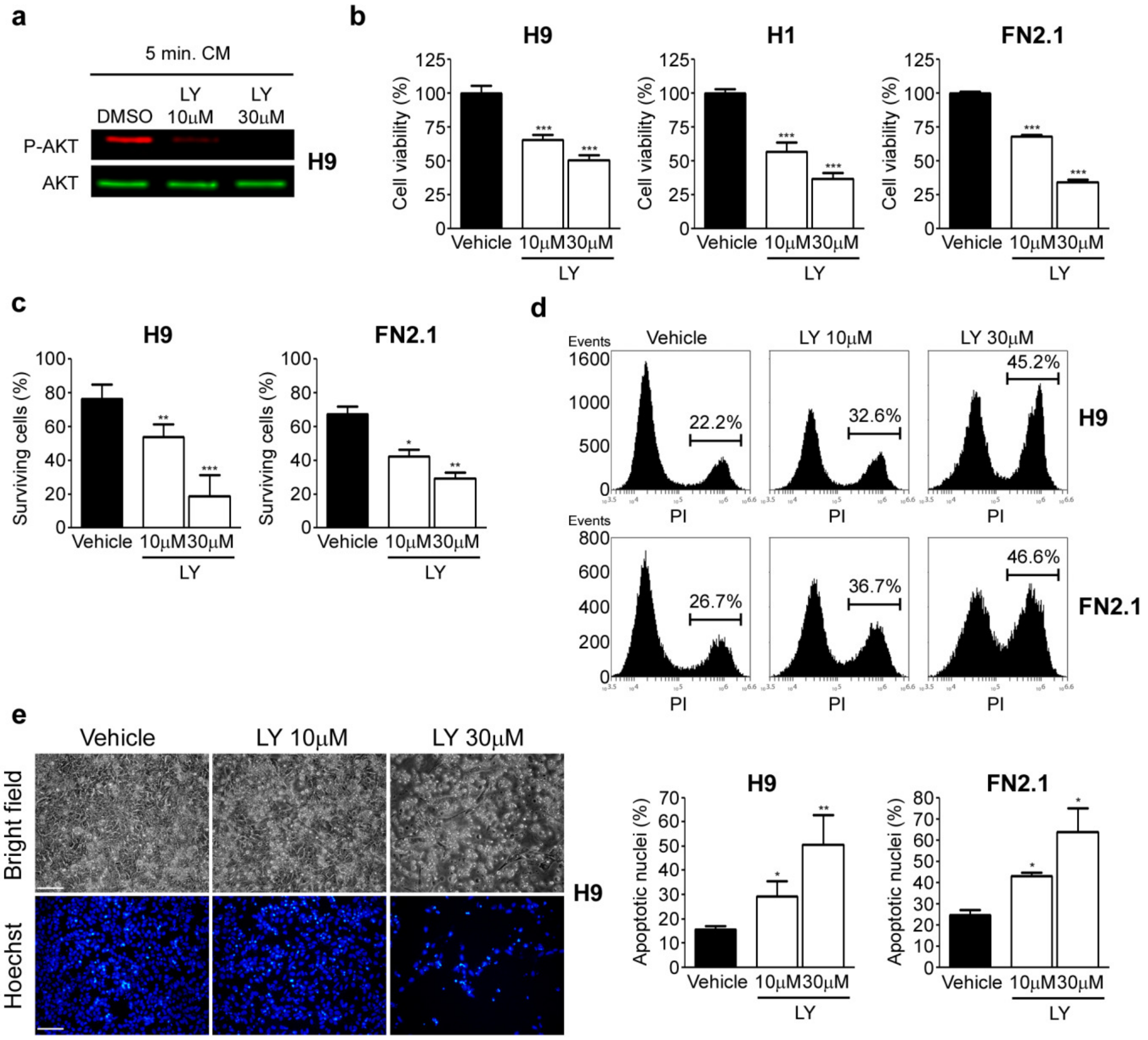

Supplementary Figure S1. ***hESCs and hiPSCs cell viability upon PI3K inhibition.*** **(a)** H9 hESCs grown on Matrigel were starved for 6 hours with KSR/bFGF-free DMEM/F12 cell culture medium and then incubated for 5 minutes in complete iMEFs conditioned medium (CM) supplemented with 8 ng/ml bFGF plus DMSO (Vehicle) or PI3K inhibitor LY294002 (LY, 10 and 30µM). After the starvation/stimulation period, p-AKT (Ser473) and AKT expression levels were analyzed and quantified by Western blots with IR fluorescence secondary antibodies and Odyssey Imagers in order to test LY inhibitor efficacy in human pluripotent stem cells. **(b)** H9, H1 hESCs and FN2.1 hiPSCs cell viability was analyzed 24 hours post-treatment with increasing concentrations of LY (10 and 30µM) by XTT colorimetric assay. Vehicle = DMSO. Mean + SEM from three independent experiments are shown. Statistical analysis was done by one-way ANOVAs followed by Tukey's multiple comparisons test, *** = $p< 0.001$ vs. Vehicle. **(c)** Histogram shows percentage of surviving cells assessed by Trypan blue exclusion method 24 hours after incubation with PI3K inhibitor LY (10 and 30µM). Mean + SEM from at least three independent experiments are shown. Statistical analysis was done by one-way ANOVAs followed by Tukey's multiple comparisons test, * $p = < 0.05$; ** $p = < 0.01$ and *** $p = < 0.001$ vs. Vehicle (DMSO). **(d)** Representative histograms, of three independent experiments, of Propidium iodide (PI) stained H9 and FN2.1 unfixed cells treated for 24 hours with LY (10 and 30µM). Percentage of PI positive cells (late apoptotic or necrotic) was determined by flow cytometric analysis. Vehicle: DMSO. **(e)** Chromatin condensation was analyzed by Hoechst staining 24 hours after incubation of H9 and FN2.1 cells with LY (10 and 30µM). Figure shows representative images (H9 cells) and means +

SEM from three independent experiments are graphed for % of apoptotic nuclei. The scale bar represents 100 µm. Statistical analysis was done by Student´s t-test, * $p = < 0.05$ and ** $p = < 0.01$ vs. Vehicle (DMSO).

hESCs and hiPSCs are insensitive to Rapamycin-mediated inhibition of mTOR

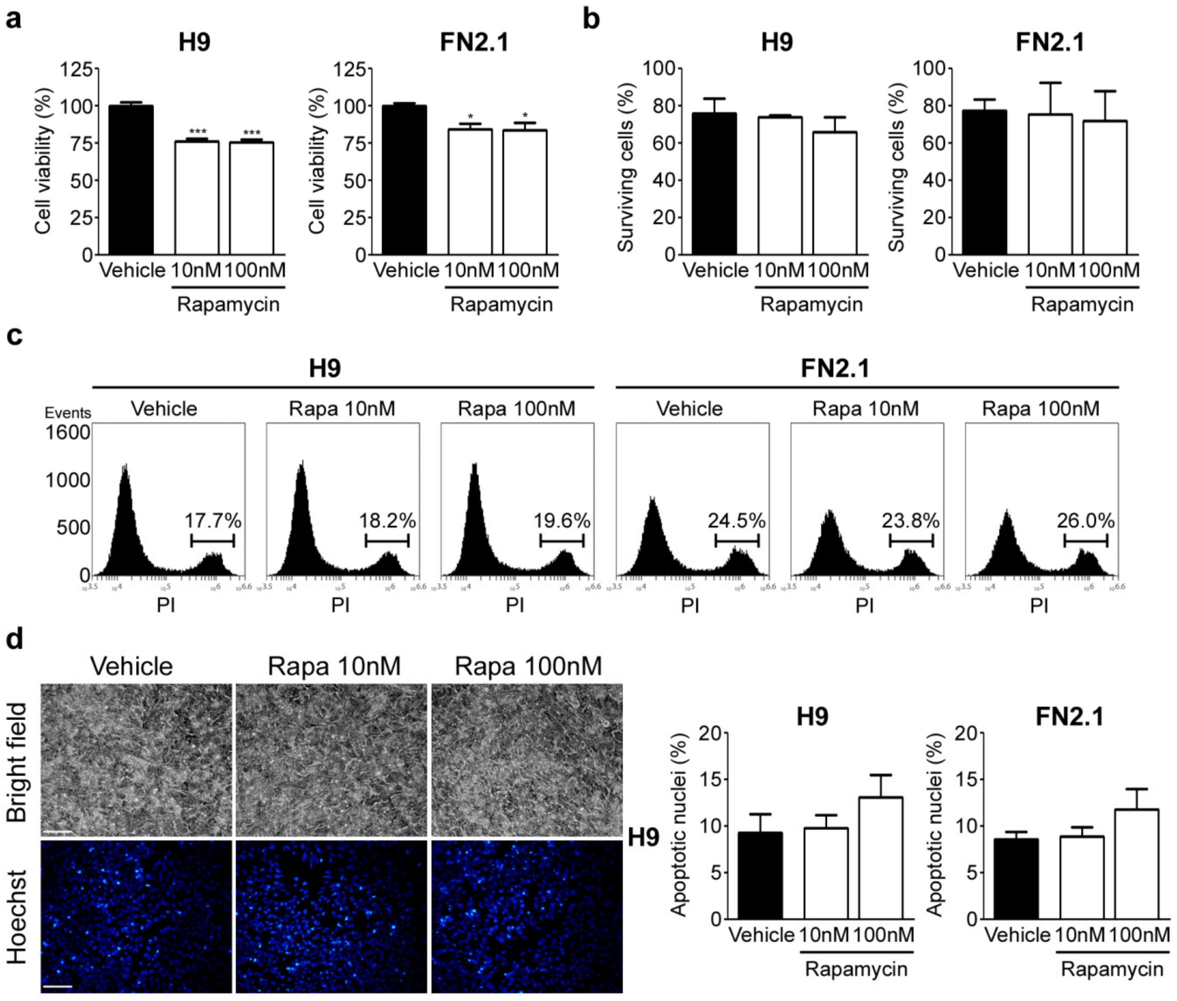

Supplementary Figure S2. ***hESCs and hiPSCs are insensitive to Rapamycin-mediated inhibition of mTOR.*** **(a)** H9 hESCs and FN2.1 hiPSCs cell viability was analyzed 24 hours post-treatment with increasing concentrations of mTOR inhibitor

Rapamycin (10 and 100nM) by XTT colorimetric assay. Vehicle = DMSO. Mean + SEM from at least three independent experiments are shown. Statistical analysis was done by one-way ANOVAs followed by Tukey's multiple comparisons test, * = $p < 0.05$ and *** = $p < 0.001$ vs. Vehicle. **(b)** Histogram shows percentage of surviving cells assessed by Trypan blue exclusion method 24 hours after incubation with mTOR inhibitor Rapamycin (10 and 100nM). Mean + SEM from three independent experiments are shown. Statistical analysis was done by one-way ANOVAs followed by Tukey's multiple comparisons test. **(c)** Representative histograms, of three independent experiments, of Propidium iodide (PI) stained H9 and FN2.1 unfixed cells treated for 24 hours with Rapamycin (Rapa, 10 and 100nM). Percentage of PI positive cells (late apoptotic or necrotic) was determined by flow cytometric analysis. Vehicle: DMSO. **(d)** Chromatin condensation was analyzed by Hoechst staining 24 hours after incubation of H9 and FN2.1 cells with Rapamycin (Rapa, 10 and 100nM). Figure shows representative images (H9 cells) and means + SEM from three independent experiments are graphed for % of apoptotic nuclei. The scale bar represents 100 µm. Statistical analysis was done by Student´s t-test.

Rapamycin induces autophagy of glioma stem cells

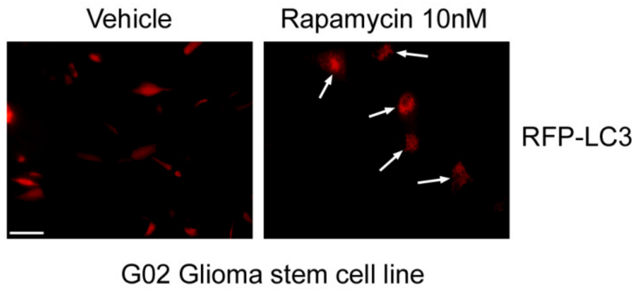

Supplementary Figure S3. ***Rapamycin induces autophagy of glioma stem cells.*** RFP-LC3-transfected G02 Glioma stem cells were treated with Rapamycin (10nM) for 24 hours in order to test Rapamycin effectiveness. Vehicle: DMSO. Representative images are shown. Arrows indicate red dots (punctuate signal), which confirmed effective autophagy induction in Rapamycin treated cells. The scale bar represents 100 µm.

Involvement of GSK3β signaling in AKT regulation of cell viability and apoptosis of hESCs and hiPSCs grown with defined media E8

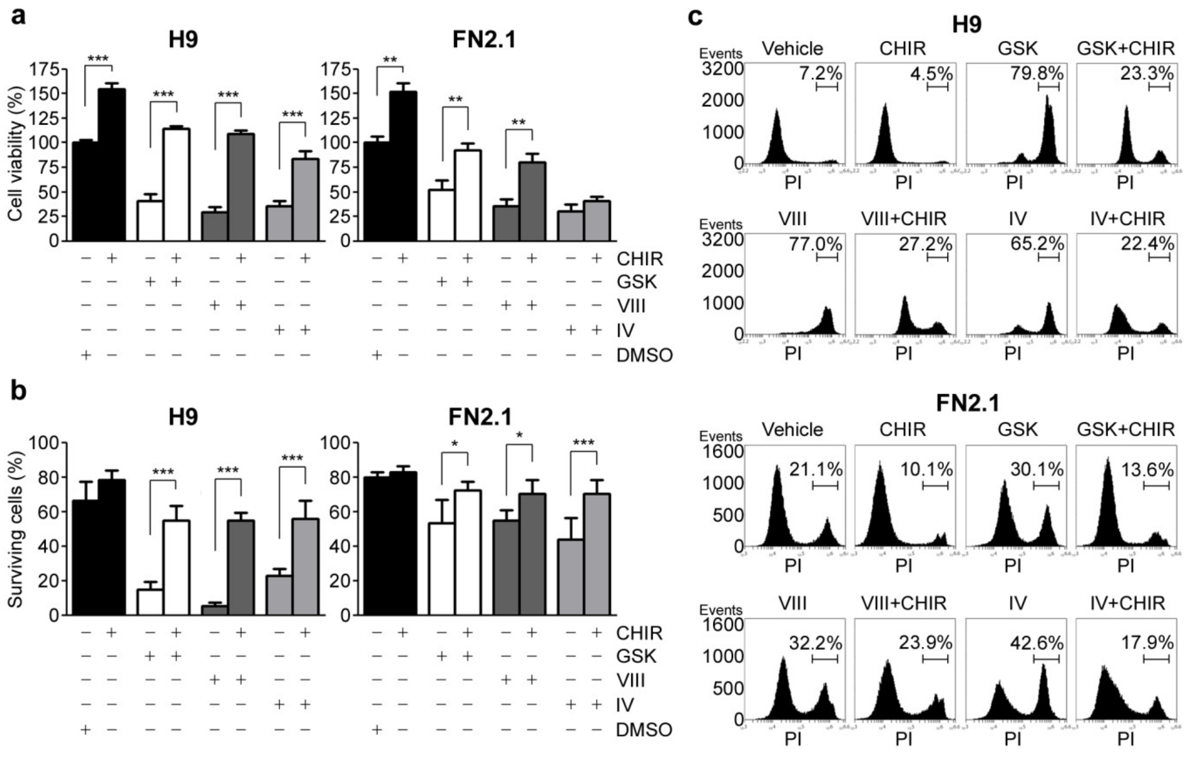

Supplementary Figure S4. ***Involvement of GSK3β signaling in AKT regulation of cell viability and apoptosis of hESCs and hiPSCs grown with defined media E8.*** H9 hESCs and FN2.1 hiPSCs were grown until confluence with E8 media in Vitronectin coated dishes and then: **(a)** Cell viability was analyzed by XTT

colorimetric assay at 24 hours post-treatment with AKT inhibitors IV (IV, 1µM), VIII (VIII, 10µM) and GSKi (GSK, 1µM) in the presence or absence of CHIRi (CHIR, 3µM). Vehicle: DMSO. Mean + SEM from three independent experiments are shown. Statistical analysis was done by one-way ANOVAs followed by Tukey's multiple comparisons test, *** = $p < 0.001$ and ** = $p < 0.01$ vs. Vehicle. **(b)** Histograms show percentage of surviving cells assessed by Trypan blue exclusion method 24 hours post-AKT inhibitors treatment [IV (1µM), VIII (10µM) and GSK (1µM)] with or without CHIRi (CHIR, 3µM). Mean + SEM from three independent experiments are shown. Vehicle: DMSO. Statistical analysis was done by one-way ANOVAs followed by Tukey's multiple comparisons test, *** = $p< 0.001$ and * = $p < 0.05$ vs. Vehicle. **(c)** Representative histograms, of three independent experiments, of Propidium iodide (PI) stained H9 and FN2.1 unfixed cells treated for 24 hours with AKT inhibitors [IV (1µM), VIII (10µM) and GSK (1µM)] in combination or not with CHIRi (CHIR, 3µM). Percentage of PI positive cells (late apoptotic or necrotic) was determined by flow cytometric analysis. Vehicle: DMSO.
